# Two Traditional Chinese Medicines *Curcumae Radix* and *Curcumae Rhizoma*: An Ethnopharmacology, Phytochemistry, and Pharmacology Review

**DOI:** 10.1155/2016/4973128

**Published:** 2016-02-18

**Authors:** Yang Zhou, Meng Xie, Yan Song, Wenping Wang, Haoran Zhao, Yuxin Tian, Yan Wang, Shaojuan Bai, Yichen Zhao, Xiaoyi Chen, Gaimei She

**Affiliations:** ^1^School of Chinese Pharmacy, Beijing University of Chinese Medicine, Beijing 100102, China; ^2^Pharmacy College, Ningxia Medical University, Ningxia 750000, China

## Abstract

*Curcumae Rhizoma*, known as Ezhu (Chinese: 莪术), and* Curcumae Radix*, known as Yujin (Chinese: 郁金), are different plant parts coming from three same species according to China Pharmacopoeia. Actually, they are used in different ways in TCM clinical treatment.* Curcumae Rhizoma* is mainly used as antitumor drug, while* Curcumae Radix* has been used as antidepressant and cholagogue.* Curcumae Rhizoma* and* Curcumae Radix* are confused in variety and source, even in clinical trials by some nonprofessional workers. So it is important for us to make them clear. This review is aimed at summarizing the ethnopharmacology, phytochemical, and pharmacological differences between* Curcumae Radix* and* Curcumae Rhizoma* by SciFinder, CNKI, and so on, to use them exactly and clearly. Further studies on* Curcumae Rhizoma and Curcumae Radix* can lead to the development of new drugs and therapeutics for various diseases on the basis of the TCM theory.

## 1. Introduction

*Curcumae Rhizoma* (Chinese: 莪术) and* Curcumae Radix* (Chinese: 郁金) are two Chinese medicines used commonly in both traditional treatment and modern clinical care. The Chinese Pharmacopoeia recorded that* Curcumae Radix* should be the dry radix of* Curcuma wenyujin* Y. H. Chen and C. Ling,* C*.* longa* L.,* C*.* kwangsiensis* S. G. Lee, and* C*.* phaeocaulis* Valeton. And* Curcumae Rhizoma* should be the dry rhizomes derived from the above-mentioned species except* C*.* longa* L [1]. They are similar in source but different in medicinal parts.

It is recorded in the TCM ancient books that their flavors are both pungent and bitter. The nature of* Curcumae Rhizoma* is warm and attributive to the liver and spleen meridians, while* Curcumae Radix* is cold and attributive to the liver, heart, and lung meridians.

*Curcumae Rhizoma* and* Curcumae Radix* have their respective priorities on TCM efficacy.* Curcumae Radix* is particularly effective in activating qi for resolving stagnation (行气解郁), clearing heart fire and cooling blood (清心凉血), together with normalizing gallbladder to cure jaundice (利胆退黄). TCM experts mainly used it to cure illnesses like jaundice, cholelithiasis caused by dampness-heat of liver and gallbladder (湿热黄疸症), and so on.* Curcumae Rhizoma* is good at treating the syndrome of food retention (食积症) and accumulation of extravasated blood (血积症). In general, they are both effective in promoting blood circulation for alleviating pain (活血止痛) and activating qi to resolve stagnation (行气消瘀), whereas on exerting drug efficacy,* Curcumae Rhizoma* is stronger than* Curcumae Radix* on the ability of invigorating the circulation of blood (活血). On this account, it is forbidden to be used in menstruation and pregnancy.

Most recent pharmacological researches on* Curcumae Rhizoma* laid emphasis on the anticancer effect particularly.* Curcumae Rhizoma* has been developed into a variety of formulations and used in clinical treatment. About* Curcumae Radix*, its reports were relatively few, and most focused on the effect of antidepressant, liver-protection, and cholagogue. Actually, they have something in common, some activities involving antitumor [2, 3], antivirus [4, 5], anti-inflammatory [6–9], and so forth; the chemical compositive contents and pharmacological activities of certain effective compounds extracted from the volatile oils were mostly focused on [10, 11].

Now, related references reviewed the traditional effect, pharmacological activities, and clinical application of* Curcumae Rhizoma* and* Curcumae Radix* [12, 13]. And they have always been reviewed, respectively. There are few to sum up between* Curcumae Rhizoma* and* Curcumae Radix* in a contrastive sight owing to their similar originals.

In the present review, we provide a systematical and comparative summary on ethnopharmacology, phytochemistry, and pharmacology of* Curcumae Rhizoma* and* Curcumae Radix*; in addition, we attempt to explore the inner link with and difference between two medicines, to well understand and use two Chinese medicines preferably. The possible tendency and perspective for future investigation of* Curcumae Rhizoma* and* Curcumae Radix* are discussed too.

## 2. Botany

*Curcumae Rhizoma* and* Curcumae Radix* are two herbal medicines of genus* Curcuma*, family Zingiberaceae.* Curcumae Rhizoma* comes from the dry rhizomes derived from only* C*.* wenyujin*,* C*.* kwangsiensis,* and* C*.* phaeocaulis*, while* Curcumae Radix* should be the dry radix from four species including above-mentioned ones and* C*.* longa*, as the Chinese Pharmacopoeia recorded.

The same source of* Curcumae Rhizoma* and* Curcumae Radix* is* C*.* wenyujin*,* C*.* kwangsiensis,* and* C*.* phaeocaulis* whose characteristics are introduced as follows. As the typical botanical characters,* C*.* wenyujin* grows to the height of 0.8–1.6 m. The leaves are blade oblong or ovate oblong with the length of 35–75 cm and the width of 14–22 cm. They are glabrous, base subrounded or broadly cuneate, apex acute, or shortly caudate. The inflorescences are on separate shoots arising from rhizomes. The leaves of* C*.* kwangsiensis* are blade elliptic-lanceolate and pubescent with the length of 14–40 cm and the width of 4.5–9.5 cm. The inflorescences are terminal on pseudostems or on separate shoots arising from rhizomes. As for* C*.* phaeocaulis*, their leaves are oblong, oval, disciform, or narrowly ovate, and inflorescences are cylindrical and spicate.

**Figure 1:**
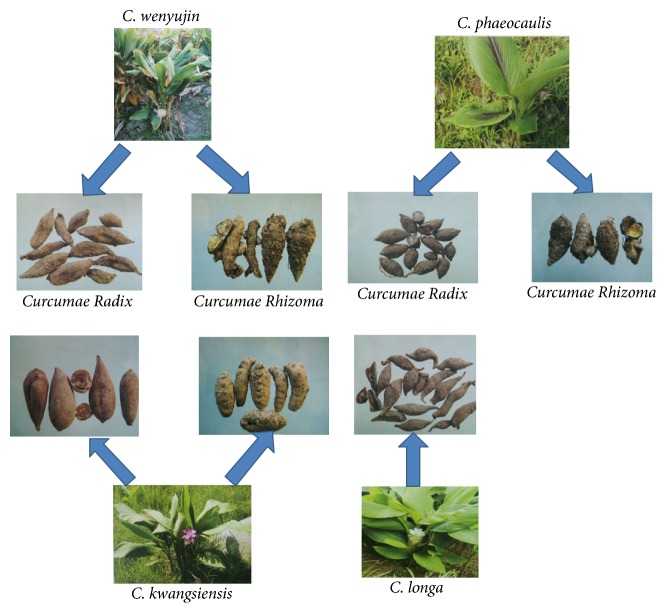
The typical botanical characters of* Curcumae Rhizoma* and* Curcumae Radix* from different sources.

*C. longa* is the source of* Curcumae Radix*. But it is not the origin of* Curcumae Rhizoma*.* C. longa* grows to the height of 1 m. The leaves are blade green, glabrous, oblong, or elliptic and the inflorescences are terminal on pseudostems. In conclusion, the radix of all 4 species mentioned above is* Curcumae Radix*. The* Rhizoma* species is known as* Curcumae Rhizoma*, excluding the plant* C. longa* (Figure 1). Generally, either* Curcumae Rhizoma* or* Curcumae Radix* is picked in winter when the stems and leaves are withered. And they should be dried before being taken into clinical application.

## 3. Ethnopharmacology

In the long development history of Chinese traditional medicine,* Curcumae Rhizoma* and* Curcumae Radix* play a significant role in the traditional treatment.* Curcumae Rhizoma* was initially recorded in Lei Gong Pao Zhi Lun during the southern and northern dynasties, which is deemed as the earliest monograph of science on processing Chinese medicine in the world. In Ri Hua Zi Ben Cao,* Curcumae Rhizoma* was recounted to stimulate one's appetite, help digestion, promote menstruation, eliminate blood stasis, and alleviate pain caused by the injuries from falls.* Curcumae Radix* was initially recorded in Yao Xing Lun, described to cure the irregularity of qi and blood and the accumulation of cold. In Kai Bao Ben Cao,* Curcumae Radix* was described to activate blood circulation, descend qi, and promote tissue regeneration, and arrest bleeding. In Compendium of Materia Medica, a monograph of TCM,* Curcumae Radix* was applied to cure the blood disease.

*Curcumae Radix* has obvious effects on syndrome of qi stagnation and blood stasis (气滞血瘀症) which may cause hematemesis (吐血), bleeding from five aperture or subcutaneous tissue (衄血), hematuria (尿血), bloody stranguria (血淋), and aberratio mensium (倒经). Also, it could cure heat disease and unconsciousness (热病神昏), epilepsy and internal stagnation of phlegm (癫痫痰闭). Furthermore,* Curcumae Radix* could be chosen to cure such patients characterized by dampness-heat of liver and gallbladder (肝胆湿热) or jaundice (黄疸) or cholelithiasis (胆石症).* Curcumae Rhizoma* does well in treating the disease mass in the abdomen accumulation (癥瘕积聚), amenorrhea (经闭), the blood stasis of heart and abdomen (心腹血瘀), dyspepsia (食积), and distending pain of gastral cavity and abdominal pain (脘腹胀痛). And the two medicines are usually used in herb pairs, such as* Curcumae Rhizoma* and* Sparganii Rhizoma*,* Curcumae Rhizoma* and* Astragali Radix*. Such medicine application is a progress in treatment which could make two medicines display advantages, respectively, and exert therapeutic effects together. Because of their fabulous and definite clinical effects, many classic prescriptions (Table 1) created by the ancient famous doctors were handed down from generation to generation through the repeated clinical verification for thousands of years.

## 4. Pharmacological Activities

The pharmacological actions of* Curcumae Rhizoma* and* Curcumae Radix* have gained much attention. Generally speaking,* Curcumae Rhizoma* has effects in antitumor, antiplatelet aggregation and antithrombosis, hepatoprotective, antioxidant, antimicrobial, and antiviral activity, and so forth. Among these, antitumor activity is most widely and emphatically studied [59, 62–64]. It played a significant role in the treatment of cancer and tumor in clinic [65, 66].* Curcumae Radix* also owns its unique priorities. In general,* Curcumae Radix* is mainly used as hepatic protectant and choleretic drug. In recent years, it has been used for treating depressive disorder with marked curative effect [67]. There are some relations between traditional effects and modern pharmacological. For example,* Curcumae Rhizoma*, the antitumor activity, and antithrombosis activity have been convinced to be relevant to the traditional effect “activating qi and breaking blood stasis.”

### 4.1. Antitumor Activity

Nowadays, the antitumor effect of the* Curcuma* species has been widely studied. And they are focused on two varieties* C. wenyujin* and* C. longa*, which are carried out not only* in vivo* but also* in vitro*. In summary, the mechanism of the antitumor activity of* Curcuma* species was mainly concentrated on the cytotoxicity and cell apoptosis-induced.

*Curcumae Rhizoma* is widely used in the traditional Chinese medicine whose essential oils are widely applied in the treatment of tumor in China. And the bioactive compounds in* Curcumae Rhizoma* can generally be divided into two categories: volatile and nonvolatile components [12]. Among the volatile compounds, mostly are monoterpenoids and sesquiterpenoids. Particularly, *β*-elemene is the most widely studied component. It has been proved to possess broad-spectrum antitumor activity and effective treatment on the several type tumors [65–68]. As for nonvolatile compounds, curcumin and polysaccharides took up a large proportion. Similar to the former, they can also cure many kinds of tumor effectively. The antitumor effects of these terpenoids are found related to the retardation of cell cycle arrest, the induction of apoptosis, and the inhibition of metastasis or tissue invasion [12]. As a kind of acknowledged main effective constituents, people found that *β*-elemene has the ability to inhibit the activity of ovarian cancer cells [65]. And the inhibition of *β*-elemene-induced cancer cell proliferation is mainly due to the apoptotic cell death and cell cycle arrest [68, 69]. Further study was conducted to clarify that *β*-elemene extracted from* C*.* wenyujin* could antagonize glioblastoma cells by inducing apoptosis [13]. And this active compound was found to exert the effects inhibiting the growth of H_22_ tumor cells time- and dose-dependently. It is worth noting that *β*-elemene affects the expression of histone H_1_ only at the protein level but it is not involved in regulation at the gene level [70].

There are also some other active chemical compounds of* Curcumae Rhizoma*. Furanodiene, germacrone, furanodiene, and 13-hydroxygermacrone, sesquiterpenes extracted from the essential oil of* Curcumae Rhizoma* (*C*.* wenyujin*), were shown to possess the inhibiting UVB-induced upregulation of the mRNA and protein expression levels of MMP-1, MMP-2, and MMP-3 in human keratinocytes [71]. It is reported that its essential oil extract has the ability to inhibit tumor growth whose treatment resulted in apoptosis in Hela cells [3].

The polysaccharides extracted from* Curcumae Rhizoma* (*C*.* kwangsiensis*) were demonstrated to significantly inhibit the proliferation of CNE-2 cells, which was possible through the induction of apoptosis mediated by attenuating Bcl-2 expression and promoting p53 expression [72]. And curcumol was evidenced to have an effect in the apoptosis in SGC-7901 [63].

Generally speaking, the amount of reports on* Curcumae Radix* is somehow less compared to* Curcumae Rhizoma*.* Curcumae Radix* confirmed that it could inhibit the appearance of the tumor on the colonic epithelium and chemical carcinogenesis in lots of clinical practice study [73]. An extracted compound, curcumrinol C, demonstrated that it could induce tumor cells apoptosis. And this result was associated with the increase on the expression of Caspase-9, Caspase-3, Caspase-7, and PARP (89 KD) [62]. By using MTT method, diterpenoid from* Curcumae Radix* was investigated to have inhibitory effects on the growth of human gastric cancer cell SGC-7901, and the mechanism might be related to the apoptosis executive protein Caspase-3 activated by regulating p65 via p38MAPK [74]. And by using RT-PCR method, the same result was obtained, but the mechanism was associated with the downregulation of VEGF expression level [64].

Curcumin extracted from* C. longa* not only has strong cytotoxic activity but also can inhibit cells by inducing apoptosis. For example, in a study, Tca8113 and Tb cells were treated with different concentrations of curcumin (6.25, 12.50, 25.00, and 50.00 *μ*mol/L), respectively, for 24 hours. The curcumin can notably inhibit proliferation and induce apoptosis of Tca8113 and Tb cells of human oral squamous cell carcinoma [75]. Now, a lot of work had been focused on the antitumor activity of curcumin. Curcumin can inhibit a variety of tumor cells' growth and prevent from skin cancer, gastric cancer, adenocarcinoma of duodenum, colon cancer, and breast cancer of rodent induced by chemical and radioactive materials. It can also significantly reduce the number and the mass of tumors [76]. Curcumin is an acknowledged active component which is a scavenger of reactive oxygen species and has properties in skin and forestomach carcinogenesis and various pharmaceutical applications [77]. Moreover, the half inhibitory concentration, IC_50_, of the extract named curcuminol E from the* Curcumae Radix* (*C*.* wenyujin*) on HL-60 was 4.2 mg/L and K562 being equal to 2.7 mg/L [59].* Curcumae Rhizoma* showed different mechanism of the antitumor activity. For example, fractionated extracts of* C*.* wenyujin* (*Curcumae Rhizoma* or* Curcumae Radix* not mentioned in original literature) were tested for their potential to modulate the MDR phenotype and function of P-gp in MCF-7/ADR and A549/Taxol cells* in vitro* [78].

### 4.2. Antiplatelet Aggregation and Antithrombosis Activities

It is reported that curdione isolated from the essential oil of* Curcumae Rhizoma* (*C. wenyujin*) using the silica gel column chromatography method has a principal and active inhibitory effect on human platelet aggregation* in vitro* and* in vivo* testing. Specifically, we found that curdione could preferentially inhibit PAF- and thrombin-induced human platelet aggregation in a concentration-dependent manner (IC_50_: 60–80 *μ*M). But its inhibitory activity on ADP- and AA-induced platelet aggregation was very weak in the* in vitro* test. Also, curdione played an important part in the inhibition of the P-selectin expression, the increase in cAMP levels, and the attenuation of intracellular Ca^2+^ mobilization in PAF-activated platelets. Moreover, curdione showed significant antithrombotic activity* in vivo* by testing in a tail thrombosis model [79]. Another compound, *β*-elemene extracted from the* Curcumae Rhizoma* (*C*.* wenyujin*), could extend coagulation time in normal mice in a dose-dependent manner. It means that, along with the increase of dosage, antithrombosis action by *β*-elemene was increased gradually. It played a role of antithrombosis activity by inhibiting platelet aggregation, releasing TXA_2_, and decreasing the production of platelet [80]. In another study, by using photo chemical reaction, *β*-elemene also proved that it may contribute to the prevention of thrombosis [81]. Moreover, in an experimental study, the* Curcumae Rhizoma* (*C*.* phaeocaulis*) revealed an active effect on inhibiting platelet aggregation and prolonging time of mice analgesic. And the products with vinegar are more powerful [82].

There are no so many researches which could prove the activity of* Curcumae Radix*. The polysaccharides from* Curcumae Radix* (*C*.* kwangsiensis*) held anticoagulant activities confirmed by prolonging significantly the clotting time (CT), thrombin time (TT), and activated partial thromboplastin time (APTT) of mice in* in vivo* study [83].

### 4.3. Hepatoprotective Activity

Through summarizing literatures, the author found that there are two extracts which have the antihepatotoxic activity. One, ethanolic extract and essential oil, is mostly extracted from* C*.* kwangsiensis* or* C*.* wenyujin*. For example, the volatile oil of* C. wenyujin* can reduce the activity of S-GDT, have a certain repair effect on liver function, and correct A/G proportion inversion caused by hepatitis. It also can protect liver cells and promote the regeneration of liver tissues by the clinical observation and animal experiment [84]. The other, mainly curcumin, is extracted from* C. longa*.

Essential oils extracted from* Curcumae Rhizoma* (*C*.* kwangsiensis*) are effective in protecting liver from the damage induced by CCl_4_, TAA significantly, and it may be due to descending transaminase function and drug enzyme-induced function [85].

The model mice of acute hepatic injury were established with intraperitoneal injecting 0.2% CCl_4_ 0.2 mL/10 mg to observe the activities of superoxide dismutase (SOD) and the content of malondialdehyde (MDA) in hepatic tissue. It convinced that* Curcumae Radix* could protect the liver from acute hepatic injured induced with CCl_4_ in mice [86]. Curcumin has powerful protective effects against acute liver injury induced by CCl_4_, D-GaLN, BCG, and LPS. And it might have therapeutical effect on liver fibrosis [87]. Furthermore, the eight sesquiterpenes, furanodiene, curdione, neocurdione, dehydrocurdione, germacrone, 13-hydroxygermacrone, curcumenol, and curcumenone, also showed a protective effect against D-GaLN/tumor necrosis factor-alpha-induced liver injury in [88]. As for hepatic toxicity caused by some things in life like alcohol (drink), paracetamol (drug) was confirmed experimentally to be treated by curcumin. Curcumin can improve antioxidant enzyme activity and protect liver from ethanol-induced oxidative stress damage.

### 4.4. Antioxidant Activity

The two medicines have antioxidant effects through different mechanisms. For example, it is observed that the medium and the highest dose of aqueous extracted from* Curcumae Rhizoma *(*C. kwangsiensis*) could increase the CAT and GSH-Px activities in cytosols in liver of rats which are recognized as the main component part of antioxidant system in the body. It is indicated that the extracts at a large dose could increase the antioxidative activity of the rats' liver [89]. The rise in free radicals level is thought to be related to the increase in TC, LDL-c, and TG and fall of HDL-c. And in a study,* Curcumae Rhizoma* (*C. kwangsiensis*) is proved to contain natural antioxidants, polysaccharides in which isoflavones and a class of phytochemicals can be found. It revealed that the polysaccharides of water extract possessed strong free radicals scavenging activities.

It can bring down the elevated levels of TC, LDL, VLDL-c, and TG in high-fat animals on protecting against oxidative injury induced by high-fat diet treatment [90]. Moreover,* Curcumae Rhizoma* oil could elevate the antioxidant enzymes in blood such as SOD, GSH-Px, and MDA [91].

As for* Curcumae Radix*, the extract of the* Curcumae Radix* (*C*.* wenyujin*) could reduce the lipid peroxidation production by protecting and improving the antioxidant activity like CuZn-SOD, Mn-SOD, GSH-Px, and CAT in rats which were damaged by radiation [92]. There are many reports on the antioxidative activity of* C*.* longa*. The PC-OOH level rising was considered as an oxidative stress marker caused by alcohol consumption. And curcumin has the power to lower the PC-OOH level in liver significantly [93]. Also, it has the DPPH radical scavenging effects with IC_50_ values of 2.8 *μ*M [94]. Curcumin can also increase the activity of SOD, which is considered to be associated with induction or progression of many diseases closely [94–96].

### 4.5. Anti-Inflammatory Effect

There are many reports indicating anti-inflammatory effect of* Curcumae Radix.* The ethanol extract and water extract of* Curcumae Radix *(*C. kwangsiensis*) possessed protective effect against dimethylbenzene and ethylic acid induced inflammation in the pinna swelling model of mice and the cotton ball granuloma model of mice. It was associated with producing significant inhibitory effects on the increased blood capillary permeability and the swollen and hyperplastic granulation tissue in rats [6]. By using hot plate test, xylene-induced mouse ear edema test, and chemical irritation-induced writhing test, we found that the ethyl acetate extract of* Curcumae Radix *(*C*.* wenyujin*) had the analgesic and anti-inflammatory effects. The extract had the analgesic effects improved with increased-dosages by physical and chemical stimulated. It had obviously inhibited inflammatory edema and celiac capillary permeability, and it could also reduce the level of TNF-*α*. The mechanisms may be inhibited by the level of TNF-*α* [7]. Similarly, another study also showed that it had stronger anti-inflammatory and antinociceptive effects [9]. Furthermore, curcumin from* C. longa* attenuated the development of allergic airway inflammation and hyperresponsiveness, possibly through inhibition of NF-*Κ*B activation in the asthmatic lung tissue [97]. In addition, polysaccharide and water extract of* C*.* longa* might be responsible for anti-inflammatory action by downregulating the PGE2 and IL-12 levels in LPS stimulated mouse splenocytes [98].

The* Curcumae Rhizoma* also has the anti-inflammatory activity. Sun et al. found that the derived sesquiterpenoids named furanodiene and curzerene extracted from* Curcumae Rhizoma* (*C*.* wenyujin*) have significant inhibition effect on the TNF-*α* factor excreted by THP-1 cells [99].

### 4.6. Antimicrobial and Antiviral Activity

Essential oils from* Curcumae Rhizoma* (*C. kwangsiensis*,* C. phaeocaulis*) have stronger and broad-spectrum antagonistic activity on 6 kinds of fungi. The hyphal cell wall disappeared and protoplast was dissolved by observation on electron microscope, which indicated that* Curcumae Rhizoma* (*C. kwangsiensis*,* C. phaeocaulis*) was the promising for biological pesticide [100].

Essential oils extracted from the* C*.* wenyujin*, which come from rhizomes and radixes, were also investigated to have antimicrobial activity on the selected microorganisms including two bacterial strains (*Propionibacterium acnes* and* Staphylococcus aureus*) and one fungal strain (*Malassezia furfur*). It showed only weak inhibitory activity against* Malassezia furfur*. Only a weak inhibitory effect was observed for oil from radix of* C*.* wenyujin*. Moreover, the study proved that the monoterpenoids have a higher inhibition activity than the sesquiterpenoids do [10]. Another study demonstrated that sesquiterpenoids from* C*.* wenyujin* showed significant* in vitro* antiviral activity against the influenza virus A (A/H1N1/Guangdong/219/2006) cells with IC_50_ values ranged from 6.80 to 39.97 *μ*M and SI values ranged from 6.35 to 37.25 by using cytopathic effect (CPE) reduction assay and measuring cytotoxicity in parallel with the determination of antiviral activity [4]. Furthermore, in recent years, many studies have begun to make directional biotransformation of the extract from Curcuma. For example, the directional biotransformation of curcumol by* Penicillium janthinellum* was studied. And the compound transformed from curcumol could have a significant antivirus activity on parainfluenza virus, respiratory spore virus, and herpes simplex virus (HSV) type I* in vitro* [101].

### 4.7. The Effect on the Stomach and the Bone

The curcumin of* C*.* longa* ethanol extracted was proved to have protective effects on stomach by making the animal gastric ulcer models with cold water stress of mouse, with alcohol induction of mouse, and with delegation at gastric pylorus cardiac orifice of rice. And the animals which were randomized into different five groups were given different dosage of curcumin. The result showed that curcumin has protective effects on the gastric mucosa injury whose mechanism was possibly related to inhibition of peroxidation reaction [102]. Similarly, by the animal experiment, the other study revealed that there was delay in gastric emptying following intragastric administration of curcumin. And it could be explained by the NOS inhibitory action [103].

Curcumin extracted from* C*.* Longa* also has favorable applied value on the clinical research. In the report by Chen et al., curcumin whose dosage was up to 8000 mg/d and whose time given to the patient was 13 months has no toxicity on human [104].

Curcumin may affect the skeletal system. At the cellular level, curcumin modulates important molecular targets: transcription factors, enzymes, cell cycle proteins, cytokines, receptors, and cell surface adhesion molecules, because many curcumin targets which were mentioned above participate in the regulation of bone remodeling. And its mechanism partially related to the inhibition of NO production which somehow is similar to what is mentioned above.

In a word, as a matter of fact, curcumin could facilitate the attenuation of tissue and cellular injury in the liver, heart, kidney, brain, and bone, which are proved to be induced by oxidation or inflammation [105].

### 4.8. Other Pharmacological Activities

What is more,* Curcuma* has been investigated to exert other pharmacological activities.* In vivo* study showed that four* Curcuma* drugs derived from* C*.* longa*,* C*.* kwangsiensis*,* C*.* phaeocaulis,* and* C*.* wenyujin* had an effect on the blood vessel. The result demonstrated that the extracts curcumin and sesquiterpenes showed NO-independent relaxation effects with almost the same intensities, while polysaccharides, in contrast, showed contraction effects [106].

Polysaccharides from rhizome, stem, and leaf of* C*.* kwangsiensis* have* vitro* fibrinolytic activities by using fractional precipitation method and fibrin plate method [107].

*Curcumae Radix* has antidepressant effect. And the active parts are in petrol fraction and ethyl acetate fraction [67]. Besides,* Curcumae Radix* has cholagogue activity [108]. Moreover, curcumin has a potent protective effect against the testicular toxicity [109]. Also, curcumin has a distinct effect to reduce the blood lipid. The animal experiments showed that curcumin could effectively reduce the total cholesterol and triacylglycerol of the serum and liver tissues and increase the activity of the related enzymes involved in lipid metabolism in plasma, while the antihyperlipidaemic effect particularly can be shown in the result of reducing blood lipid and raising the level of HDL in serum [110].

### 4.9. Drug Development

In the present clinical practice, those classic prescriptions have been employed in a more extensive and intensive way. Nowadays, many prescriptions (Table 2) were applied into the clinic in the forms of pill, granule, capsule, and injection for the sake of convenience. Similarly, their effects evidenced by clinical reports are still beyond all doubt. Zedoary turmeric oil and glucose injection was used to treat 85 patients with acute upper respiratory infection and 83.5% was effective [111]. Curcuma oil injection can be also effective and shorten the duration of the main symptoms on the treatment of acute upper respiratory tract infection [112]. As for* Curcumae Radix*, for example,* Curcumae Radix* injection has been proved to be effective in curing icteric virus hepatitis [113].

## 5. Chemical Constituents of* Curcumae Rhizoma* and* Curcumae Radix*

To date, the chemical constituents from* Curcuma Rhizoma* and* Curcuma Radix* reported about** 207** compounds, mostly assigned to sesquiterpenes and diarylheptanoids, also including alkaloids, polysaccharides, and some other types. The two main types of compounds are considered to be their bioactive chemical constituents. Sesquiterpenes, also the main constituents on essential oils, are responsible for their extraordinary anticancer, anti-inflammatory, antiviral, neuroprotective, and antithrombotic effect [10, 85, 110]. Diarylheptanoids, which also possess various pharmacologic activities, such as curcumin, demethoxycurcumin, and bisdemethoxycurcumin, are the major nonvolatile compounds in* Curcumae Rhizoma* and* Curcumae Radix* [114]. In this section, we summarize and classify all of the reported constituents in* Curcumae Rhizoma* and* Curcumae Radix* from four species (*C. wenyujin*,* C*.* longa*,* C*.* kwangsiensis,* and* C*.* phaeocaulis*) that Chinese Pharmacopoeia regulated. Their structures are shown in the following, while the corresponding plant sources and references are collected in Table 3.

### 5.1. Sesquiterpenes (Figure 2)

Sesquiterpenes are defined as 15-carbon compounds formed from 3 isoprenoid units. Sesquiterpenes, extremely diverse, heterogeneous, and large group of natural compounds, are widely used in folk medicines, health-supporting preparations, and cosmetics [115]. 133 sesquiterpenes have been found in, including 53 guaiane types, 31 germacrane types, 15 bisaborane types, 12 eudesmane types, 10 elemane types, 2 spironolactone types, 4 carabrane types, 1 xanthane type, and 5 other types. These compounds are mostly reported from* Curcumae Rhizoma* of* C. wenyujin* and* C. kwangsiensis*, while two other plant species possess few. Nearly every kind of sesquiterpenes consists of* Curcumae Rhizoma*, but* Curcumae Radix* is only found in guaiane type, germacrane type, bisaborane type, eudesmane type, and elemane type. Moreover, the compounds extracted from* Curcumae Radix* are too little.

#### 5.1.1. Guaiane Type

53 sesquiterpenes with the same skeleton as the guaiane type have been isolated one by one; see compounds** 1**–**53**. This kind is a skeleton combined by a five-membered ring and seven-membered ring. Curcumol is one of the typical symbols, which is an active compound in the treatment of tumor specially. In 1965, for the first time, a Japanese scholar named Hiroshi Hikino isolated this compound identified as curcumol from the volatile oil of* C. zedoaria* Roscoe [116].* C. zedoaria Roscoe* is considered as* Curcumae Rhizoma*, which is mainly distributed in Asian countries including India, Vietnam, Japan, and China [117], but it is not the official recognitory species. Curcumol widely exists in* Curcuma* species.

#### 5.1.2. Germacrane Type

From 1966 to 1967, Hikino et al. [118] isolated a sesquiterpene named curdione firstly whose nuclear parent was a ten-membered ring. Such a large ring is flexible and easy to distort. Most compounds with this nuclear parent have many conformations under normal temperature which attaches great degree of difficulty to identify its absolute configuration for researchers. Later they achieved the aim by using the single crystal diffraction method combined with the calculation of molecular energy and the molecular model. See compounds** 54**–**84**.

#### 5.1.3. Bisaborane Type

In 1987, Uehara et al. [119] isolated 3 chemical compounds from* C*.* xanthorrhiza* and identified them, respectively, as bisacurone, bisacumol, and bisacurol.* C*.* xanthorrhiza* is used in Indonesian folk medicine with many activities, which may be used as* Curcumae Rhizoma*, while it is not included in official species [120]. Since then, 15 sesquiterpenes (**85**–**99**) of this kind had been reported by other studies one after another.

#### 5.1.4. Eudesmane Type

The structure combined with two six-membered rings is its structure characteristic (**100**–**111**). In 1967, Hiroshi Hikino first isolated curcolone and identified its absolute configuration [121].

#### 5.1.5. Elemane Type

In 1968, zedoarone was isolated from* C*.* zedoaria* Roscoe by Hikino et al. [122]. Later, other researchers found 12 sesquiterpenes (**112**–**121**) of this kind from* Curcumae Rhizoma* and* Curcumae Radix*.

### 5.2. Diarylheptanoids (Figure 3)

There is another important active ingredient named diarylheptanoids, also called curcumin components whose pharmacological activity has been one of the research hotspots in the field of medicine in* Curcumae Rhizoma*. Among them, the curcumin has been regarded as the third generations of anticancer drug to be studied by the National Cancer Institute. The nuclear parent of this kind is linear diarylheptanoids which can be divided into phenolic and nonphenolic according to the differences in substituents. Up to now, more than 10 curcumin components have been isolated from* Curcumae Rhizoma*, among which, curcumin, demethoxycurcumin, and bisdemethoxycurcumin are mostly common. 90 compounds (**133**–**190)** of this kind are all isolated from* Curcumae Rhizoma*. As for* Curcumae Radix*, there is no report.

### 5.3. Other Compounds

**Figure 4:**
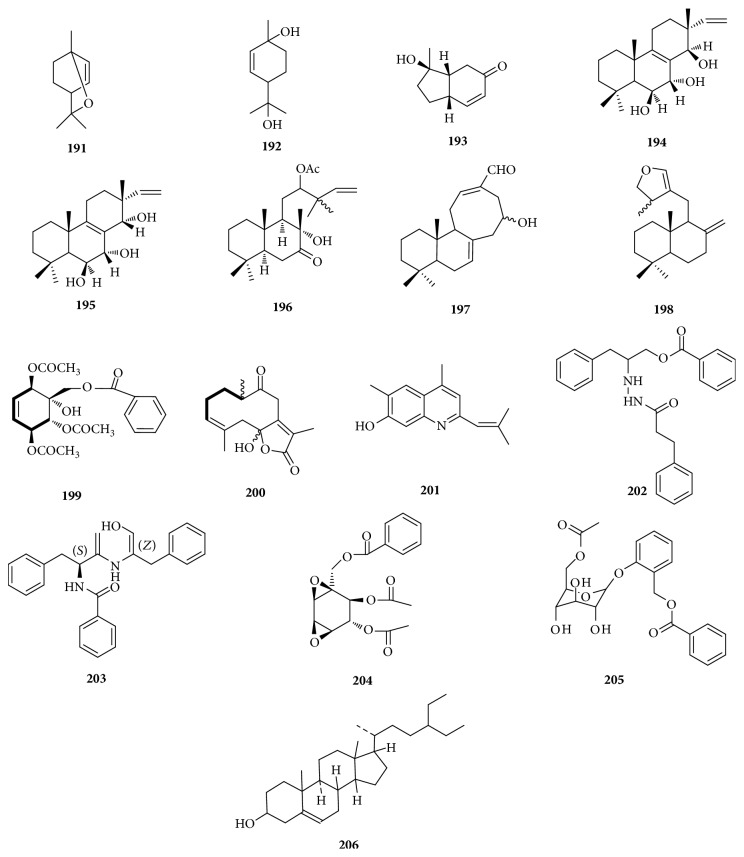
The structures of other compounds from* Curcumae Rhizoma* and* Curcumae Radix*.

Three components (**190**–**193**) are identified as monoterpene. And among all the diterpenes (**194**–**200**), two new diterpenes (**197**,** 198**) are reported from the radix of* C. wenyujin* and showed a good antitumor activity [59]. In addition, a new compound belonging to quinoline alkaloid was found in the radix of* C. longa*. Phenolics, steroid, cyclohexene epoxides, and amides are also found in* Curcumae Radix* and* Curcumae Rhizoma* (Figure 4). There are also many microelements, such as Zn, Mn, Fe, Mg, Mn, Cr, Cu, and Ca [123].

In conclusion, in comparison to* Curcumae Rhizoma*, the number of studies on* Curcumae Radix* is less. There are only 10 articles investigating chemistry of* Curcumae Radix*.

The first study on* Curcumae Rhizoma* was the study on the variety* C. phaeocaulis*, but somehow nowadays there are few studies on it. Actually, most studies on the* C. longa* focus on the rhizome. But according to one paper [25], it indicated clearly that the rhizome and radix of* C. longa* were traditionally used as two Chinese medicines, respectively, named* Jianghuang* and* Yujin* in Chinese.

## 6. Toxicity

Although* Curcumae Rhizoma* and* Curcumae Radix* have long been used in medicine clinics, systematic safety and toxicity evaluations are rare until now.

Several experiments were conducted to explore the acute toxicity and genetic toxicity of* Curcumae Rhizoma* but found to be nontoxic [58, 60]. The extract curcumol as a principle component of* Curcumae Rhizoma* is proved to be no obvious lethal effect through the long-term toxicity experiment. Different dosage groups at 1.16 g/20 mL, 2.32 g/20 mL, and 4.64 g/20 mL Tween 80, respectively, were set up, and the effect of the extracts to the rat's kidney was found to be small at low and middle but have a certain toxicity at high dose. Generally speaking, it could be used safely at low and middle dosage [124]. It is reported that* Curcumae Rhizoma* is harmful to reproductive system to a certain extent. It could decrease the rate of pregnant of female mice, increase the rate of teratogenicity, excite uterine smooth muscle, and reduce the weight of mice's testis and seminal vesicle.* Curcumae Rhizoma* is pregnancy-contraindicated drug which should be used with caution [125, 126].

The LD_50_ of mice was found to be different in sources of* Curcumae Radix*.* C. wenyujin* is the most serious [127], in which LD_50_ is 80.98 g/kg. It is equal to 485.9 times the dose of the maximum clinical dosage of adults, which demonstrated that it is safe in clinical medicine usage [128].

## 7. Quality Control

As described previously, the four species scatter across the south and southwest of China, determined to the various differences among every producing area. So the variety of them makes it difficult for us to control its quality. In the Pharmacopoeia of People's Republic of China, there are two marker components, the essential oil [no less than 1.5% (mL/g)] and germacrone (as control), chosen to control the quality of* Curcuma Rhizoma*. When it comes to* Curcuma Radix*, its standard crude drug is regarded as examine item. And thin layer chromatography (TLC) was used to get the result for the two medicines [129]. However, due to the complex constituents and multifarious pharmacological activities, the test methods mentioned above might be insufficient and inconvenient to fully illustrate the quality of them. Nowadays, to evaluate the quality of the two medicines more accurately, many ways of qualitative identification and determination of content are developed, such as twice development TLC [129], high performance liquid chromatography (HPLC) [130], fingerprinting [131], GC-MS, and pressurized liquid extraction (PLE) [132]. Among these, fingerprinting is the most widely applied. Also it is a tendency that multimethods using jointly are more rapid and reliable, such as UPLC/QTOF/MS method [133], HPLC-UV-MS [134], GC-MS fingerprinting, and HPLC fingerprinting [135]. Generally speaking, for* Curcuma Rhizoma*, curcumin is regarded as one of the effective constituents, germacrone chosen as the characteristic component, while the essential oil is the effective part. These three marker items have significant differences from variety to variety of* Curcuma Rhizoma*. As for* Curcuma Radix*, the marker components are the same with* Curcuma Rhizoma*; otherwise, curdione may also be recommend to be the standard of its quality control [136].

We are still exploring the better ways to make the quality control of* Curcuma Rhizoma* and* Curcuma Radix* more rapid and reliable. And we should also extend the studying filed of quality control, not only limited to the present methods and active components.

## 8. Concluding Remarks

In addition to the four official species,* C. wenyujin*,* C. longa*.* C. kwangsiensis*, and* C. phaeocaulis*, there are also several other varieties which are misled as* Curcumae Rhizoma* and* Curcumae Radix*. They are* C*.* Zedoaria*,* C*.* Aeruginosa*,* C*.* Aromatic,* and* C*.* xanthorrhiza* [115, 134–137] which are also been used as* Curcumae Rhizoma* or* Curcumae Radix* or other medicines. But it is not recoreded in the Chinese Pharmacopoeia.* Curcumae Rhizoma* and* Curcumae Radix*, two well-known and widely used Chinese medicines of the family* Zingiberaceae,* have been traditionally used for thousands of years. Although they both have effect in invigorating the circulation of blood,* Curcumae Rhizoma* and* Curcumae Radix* have their own respective features and priorities. However, the studies of them are disproportionate on variety and source. The traditional uses have been increasingly confirmed by hundreds of pharmacological studies* in vitro* and* in vivo*. Particularly, the antitumor studies of these two medicines are becoming more and more popular. Compared to* Curcumae Rhizoma*, the range of* Curcumae Radix* applying on this aspect is, respectively, small which only limits the treatment of gastric cancer. In most cases, there is no difference between the two medicines. Although studies on the pharmacological effects and their mechanisms have been performed, yet further study is still urgently needed to gain a better understanding of the differences between the two medicines in order to provide better service for clinical care.

Besides, most of the extracted chemical compositions have been found in the rhizoma of* C*.* wenyujin* and* C*.* longa*. So we should devote ourselves more into the chemical constituents' study of other species and focus more on* Curcumae Radix*.

In addition, recently, fingerprinting has been the most popular way to describe the chemical constituents and to control the quality of* Curcumae Rhizoma* and* Curcumae Radix*. But we still need to explore better ways to make the quality control more rapid and reliable.

Single herbs usually exert their efficacy in the form of prescriptions in TCM clinic. But the research of Chinese herbal prescriptions is someway complex, which makes it difficult to draw accurate conclusions and reflects the role of the single herb. And the study of single herb is difficult to reflect its true role in the TCM prescriptions. So we suggest that we could study* Curcumae Rhizoma* and* Curcumae Radix in* couplet medicines, such as* Curcumae Radix* and* Sparganii Rhizoma*,* Curcumae Rhizoma* and* Sparganii Rhizoma* which is similar in effect or clinical application and so on. But during study, we still should not ignore how the couplet medicines play an important part in the Chinese herbal compound prescriptions.

At last, through reading plenty of documents, we have found a problem. In recent years, many new reports have reported a large number of new compounds, but the relevant reports of their pharmacological activities are very less. And we still could not find a quantitative evaluation criterion which is exclusive and accurate enough to quality control these two medicines. So we should study further the discovery and identification of new components and medicinal efficacious composition, pharmaceutical activity screening, and evaluation and find more model of pharmacology of these two medicines. In a word, more experiments including* in vitro*,* in vivo,* and clinical studies should be encouraged to be put into effect to make us understand why* Curcumae Rhizoma* and* Curcumae Radix* are effective in more detail so as to make contribution to Chinese medical career.

## Figures and Tables

**Figure 2:**
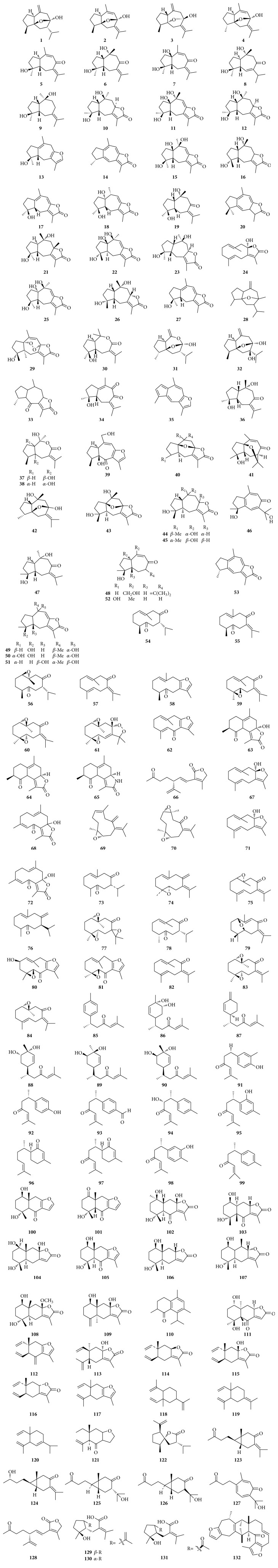
The structures of sesquiterpenes from* Curcumae Rhizoma* and* Curcumae Radix*.

**Figure 3:**
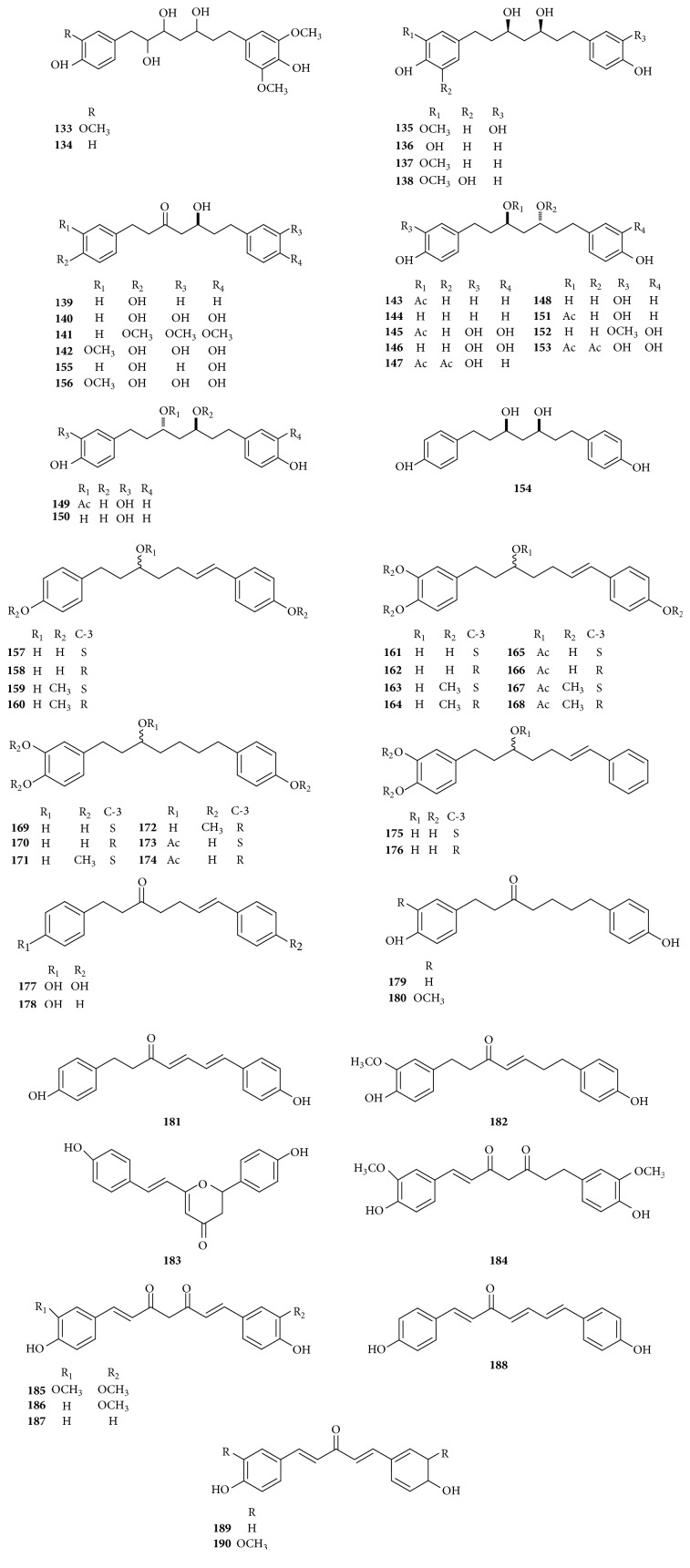
The structures of diarylheptanoids from* Curcumae Rhizoma* and* Curcumae Radix*.

**Table 1:** Classic prescriptions of *Curcumae Radix* and *Curcumae Rhizoma* in traditional and clinical usages.

Number	Preparation name	Formulation	Main compositions	References
*Yujin*				
(a) Syndrome of qi stagnation and blood stasis (气滞血瘀症)				
1	Dian Dao Mu Jin San	Powder	*Curcumae Radix*, *Aucklandiae Radix*	Yizong Jinjian
2	Xuan Yu Tong Jing Tang	Decoction	*Curcumae Radix*, *Bupleuri Radix*, *Gardeniae Fructus*, *Angelicae Sinensis Radix*, *Chuanxiong Rhizoma*, *Paeoniae Radix Alba*, *Moutan Cortex*, *Sinapis Semen*, *Rhizoma Cyperi*, *Scutellariae Radix*, *Glycyrrhizae Radix et Rhizoma*	Fuqing Zhunvke
3	Yu Jin Yin Zi	Decoction	*Curcumae Radix*, *Scutellariae Radix*, *Paeoniae Radix Rubra*, *Aurantii Fructus*, *Rehmanniae Radix*, *Arecae Pericarpium*	Shenghui
4	Chen Sha Yi Li Jin Dan	Pill	*Curcumae Radix*, *Aconiti Lateralis Radix Preparata*, *Zingiberis Rhizoma*	Qixiao Liangfang
5	Yu Jin Chuan	Powder	*Curcumae Radix*, *Arisaematis Rhizoma*, *Sparganii Rhizoma*, *Curcumae Rhizoma*, *Pinelliae Rhizoma*, *Realgar*, *Typhae Pollen*, *Paeoniae Radix Rubra*, *Faeces Trogopterori*	Chuan Yabu
6	Yu Jin Wan	Pill	*Curcumae Radix*, *Faeces Trogopterori*, *Corydalis Rhizoma*, *Realgar*, *Alumen*, *Aucklandiae Radix*, *Amomi Fructus*	Shazheng Quanshu
(b) Hematemesis (吐血), bleeding from five apertures or subcutaneous tissues (衄血), aberratio mensium (倒经), hematuria (尿血), and bloody stranguria (血淋)				
7	Sheng Di Huang Tang	Decoction	*Curcumae Radix*, *Rehmanniae Radix*, *Moutan Cortex*, *Gardeniae Fructus*	Yixue xinwu
8	Yu Jin San	Powder	*Curcumae Radix*, *Rehmanniae Radix*, *Cirsii Herba*	Pu Jifang
		Powder	*Curcumae Radix*, *Semen Nelumbinis*	Shengji Zonglu
		Powder	*Curcumae Radix*, *Sophorae Flos*	Zabing Yuanxi Xizhu
9	Yu Jin Si Wu Tang	Decoction	*Curcumae Radix*, *Rehmanniae Radix*, *Angelicae Sinensis Radix*, *Chuanxiong Rhizoma*, *Paeoniae Radix Alba*	Guanju Fangyaobu
10	Yu Jin Huang Lian Wan	Pill	*Curcumae Radix*, *Scutellariae Radix*, *Coptidis Rhizoma*, *Rhei Radix et Rhizoma*, *Talcum*, *Pharbitidis Semen*, *Poria*, *Ambrum*	Xiu Zhen
(c) Heat disease and unconsciousness (热病神昏), epilepsy and internal stagnation of phlegm (癫痫痰闭)				
11	Chang Pu Yu Jin Tang	Decoction	*Curcumae Radix*, *Gardeniae Fructus*, *Acori Tatarinowii Rhizoma*	Wenbing Quanshu
12	Bai Jin Wan	Pill	*Curcumae Radix*, *Alumen*	Shesheng Zhongmiaofang
13	Yu Jin Dan	Pill	*Curcumae Radix*, *Saposhnikoviae Radix*, *Chuanxiong Rhizoma*, *Gleditsia Officinalis*, *Alumen*, *Scolopendra*	Danxi Xinfa Fuyu
		Pill	*Curcumae Radix*, *Platycodonis Radix*, *Arisaematis Rhizoma*, *Crotonis Fructus*	Youyou Xinshu
14	Yu Jin Wan	Pill	*Curcumae Radix*, *Cinnabaris*, *Alumen*	Leizheng Zhicai
15	Yu Jin Shi Lian Zi Yin	Decoction	*Curcumae Radix*, *Moutan Cortex*, *Rehmanniae Radix*, *Ophiopogonis Radix*, *Corydalis Rhizoma*, *Caesalpiniae Semen*, *Typhae Pollen*, *Lycopi Herba*, *Poria cum Radix Pini*	Chensuan Fuke Bujie
16	Yu Jin Tang		*Curcumae Radix*, *Rehmanniae Radix*, *Anemarrhenae Rhizoma*, *Asini Corii Colla*, *Arctii Fructus*, *Armeniacae Semen Amarum*, *Platycodonis Radix*, *Glehniae Radix*, *Cicadae Periostracum*	Zabing Yuanliu Xizhu
(d) Dampness-heat of liver and gallbladder (肝胆湿热), jaundice (黄疸), and cholelithiasis (胆石症)				
17	Yu Jin Tiao Jiu San	Powder	*Curcumae Radix*, *Scutellariae Radix*, *Rhei Radix et Rhizoma*, *Saposhnikoviae Radix*, *Gardeniae Fructus*, *Angelicae Sinensis Radix*, *Chuanxiong Rhizoma*, *Paeoniae Radix Rubra*, *Gentianae Radix et Rhizoma*	Yinhai Jingwei

*Ezhu*				
(a) Mass in the abdomen accumulation (癥瘕积聚), amenorrhea (经闭), and the blood stasis of heart and abdomen (心腹血瘀)				
18	E Zhu San	Powder	*Curcumae Rhizoma*, *Sparganii Rhizoma*, *Angelicae Sinensis Radix*, *Cyperus rotundus*, *Corydalis Rhizoma*, *Paeoniae Radix Rubra*, *Aurantii Fructus*, *Rehmanniae Radix Praeparata*, *Citri Reticulatae*, *Pericarpium Virdie*, *Atractylodis Macrocephalae Rhizoma*, *Scutellariae Radix*, *Foeniculi Fructus*, *Amomi Fructus*, *Resina Toxicodendri*, *Carthami Flos*, *Chuanxiong Rhizoma*, *Glycyrrhizae Radix et Rhizoma*	Shoushi Baoyuan
19	E Zhu San	Powder	*Curcumae Rhizoma*, *Glycyrrhizae Radix et Rhizoma*, *Angelicae Sinensis Radix*, *Chuanxiong Rhizoma*, *Paeoniae Alba Radix*, *Rehmanniae Radix Praeparata*, *Foeniculi Fructus*, *Angelicae Dahuricae Radix*	De Xiao
20	Peng E Zhu San	Powder	*Curcumae Rhizoma*, *Aucklandiae Radix*	Pu Jifang
21	E Zhu Tang	Decoction	*Curcumae Rhizoma*, *Sparganii Rhizoma*, *Carthami Flos*, *Achyranthis Bidentatae Radix*, *Sappan Lignum*	Zhulin Nvke
(b) Dyspepsia (食积), distending pain of gastral cavity and abdominal pain (脘腹胀痛)				
22	E Zhu Wan	Pill	*Curcumae Rhizoma*, *Sparganii Rhizoma*, *Rhizoma Cyperi* *Arecae Semen*, *Aucklandiae Radix* *Semen Pharbitidis*. *Citri Reticulatae Pericarpium Virdie*, *Litseae Fructus*, *Aristolochiae Radix*, *Oryzae Fructus*, *Caryophylli Flos*	Zhengzhi Zhunsheng
23	E Zhu Kui Jian Tang	Decoction	*Curcumae Rhizoma*, *Carthami Flos*, *Cimicifugae Rhizoma*, *Evodiae Fructus*, *Glycyrrhizae Radix et Rhizoma*, *Bupleuri Radix*, *Alismatis Rhizoma*, *Citri Reticulatae*, *Pericarpium Virdie*, *Citri Reticulatae*, *Pericarpium*, *Scutellariae Radix*, *Magnoliae Officinalis Cortex*	Jiyang Gangmu
			*Coptidis Rhizoma*, *Alpiniae Oxyphyllae Fructus*, *Alpiniae Katsumadai*, *Semen*, *Pinelliae Rhizoma*, *Angelicae Sinensis Radix*, *Medicated Leaven*	
24	San Leng E Zhu Tang		*Curcumae Rhizoma*, *Sparganii Rhizoma*, *Bupleuri Radix*, *Pinelliae Rhizoma*, *Arecae Pericarpium*, *Gentianae Macrophyllae Radix*, *Rhizoma Cyperi*, *Citri Reticulatae*, *Pericarpium*, *Citri Reticulatae Pericarpium Virdie*, *Perillae Folium*, *Aristolochiae Radix*, *Aurantii Fructus*, *Arecae Semen*, *Glycyrrhizae Radix et Rhizoma*	Pu Ji Fang

Appendix: the literature in the form.

Wuqian (*吴谦*), 1742. Yizong Jinjia (医宗金鉴).

Fushan (傅山), 1827. Fuqing Zhunvke (*傅青主*女科).

Wangyou (王佑), 978. Shenghui (太平*圣惠*方).

Dong Suyuan (*董宿原*), 1470. Qixiao Liangfang (*奇效良*方).

Lu Zhao (*鲁照*), 1759. Chuan Yabu (*串雅补*).

Cheng Guopeng (*程国彭*), 1732. Yixue xinwu (医学心悟).

Zhu Su (朱橚), 1406. Pu Jifang (*普济*方).

Zhaojie (*赵佶*), 1161. Shengji Zonglu (*圣济总录*).

Shen Jinao (沈金鳌), 1773. ZabingYuanxi Xizhu (杂病*源洗犀烛*).

Danbo Yuanjian (丹*波元简*), Japan, year 1857. Guanju Fangyaobu (观聚方*要补*).

Li Heng (*李恒*), Ming Dynasty, year 1391. Xiu Zhenfang (*袖珍*方).

Shi Yiren (*时逸*人), Qing Dynasty. Wenbing Quanshu (温病全书).

Zhang Shiche (*张时彻*), 1550. Shesheng Zhongmiaofang (*摄生众妙*方).

Fang Guang (方广), 1536. Danxi Xinfa Fuyu (丹溪心*法附余*).

Liu Fang (刘昉), 1150. Youyou Xinshu (*幼幼新*书).

Lin Peiqin (林*佩琴*), 1851. Leizheng Zhicai (类证*治裁*).

Chen Qi (*陈沂*), Song Dynasty. Chensuan Fuke Bujie (陈素*庵妇*科补解).

Shen Jinao (沈金鳌), 1773. Zabing Yuanliu Xizhu (杂病源流*犀烛*).

Authorless, Ming Dynasty. Yinhai Jingwei (银海*精微*).

Gong Tingxian (*龚廷*贤), Ming Dynasty. Shoushi Baoyuan (寿世*保元*).

Wei Yilin (*危亦*林), 1345. Shiyi Dexiaofang (世医得效方).

Yegui (*叶桂*), Qing Dynasty. Zhulin Nvke (竹林女科).

Wang Kentang (王*肯堂*), 1602. Zhengzhi Zhunsheng (证*治准绳*).

Wu Zhiwang (*武之望*), 1626. Jiyang Gangmu (济阳纲目).

**Table 2:** Preparation approved by China Food and Drug Administration (CFDA).

Number	Preparation name	Formulation	Main compositions	Clinical uses
*Ezhu*				
1	E Zhu You Pu Tao Tang Zhu She Ye	Injection	*Curcumae Rhizoma*	Treating children viral pneumonia
2	E Zhu You Zhu She Ye	Injection	*Curcumae Rhizoma*	Curing Candida vulvovaginal disease and senile vaginitis
3	Fu Fang E Zhu You	Suppository	*Curcumae Rhizoma*, *Borneolum Syntheticum*	Curing cold caused by viruses, upper respiratory tract infection, children viral pneumonia, gastrointestinal tract ulceration, viral hepatitis A, children virus enteritis, and viral myocarditis, cerebritis, and so forth
4	Fu Fang E Zhu You Ruan Jiao Nang	Soft capsule	*Curcumae Rhizoma*, *Citri Reticulatae Pericarpium*	Activating qi for removing blood stasis, removing food retention for relieving pain, indicated for stagnation of qi and blood stasis, stomachache, and poor appetite caused by food stagnation
5	E Zhu You	Injection	*Curcumae Rhizoma*	Curing traumatic injury, the soup and fire burned, bleeding caused by the knife wound, and mosquito bites
6	Dan E Fu Kang Jian Gao	Decoction	*Curcumae Rhizoma*, *Bupleuri Radix*, *Salviae Miltiorrhiae Radix et Rhizoma*, *Notoginseng Radix et Rhizoma*, *Paeoniae Radix Rubra*, *Angelicae Sinensis Radix*, *Sparganii Rhizoma*, *Rhizoma Cyperi*, *Corydalis Rhizoma*, *Glycyrrhizae Radix et Rhizoma*	Promoting blood circulation for removing blood stasis, dispersing stagnated liver qi for regulating qi-flowing, regulating menstruation for relieving pain, and softening hard masses for eliminating abdominal mass, indicated for women irregular menstruation, dysmenorrhoea, mass in the abdomen accumulation, and endometriosis of pelvis caused by stagnation of blood stasis
7	Dan E Fu Kang Ke Li	Granule	*Curcumae Rhizoma*, *Bupleuri Radix*, *Salviae Miltiorrhizae Radix et Rhizoma*, *Notoginseng Radix et Rhizoma*, *Paeoniae Radix Rubra*, *Angelicae Sinensis Radix*, *Sparganii Rhizoma*, *Rhizoma Cyperi*, *Corydalis Rhizoma*, *Glycyrrhizae Radix et Rhizoma*	Promoting blood circulation for removing blood stasis, dispersing stagnated liver qi for regulating qi-flowing, and regulating menstruation for relieving pain, indicated for irregular menstruation, dysmenorrhoea, and mass in the abdomen accumulation
8	Huang E Jiao Nang	Capsule	*Curcumae Rhizoma*, *Persicae Semen*, *Rhei Radix et Rhizoma*, *Astragali Radix*, *Poria*, *Coicis Semen*, *Prunellae Spica*, *Leonuri Herba*, *Cinnamomi Cortex*, *Menispermi Rhizoma*, *Platycodonis Radix*, *Cyathulae Radix*.	Benefiting qi for activating blood circulation, eliminating dampness and heat Curing hyperplasia of prostate

*Yujin*				
9	Yu Jin Yin Xie Pian	Pill	*Curcumae Radix*, *Gentianae Macrophyllae Radix*, *Curcumae Rhizoma*, *Angelicae Sinensis Radix*, *Persicae Semen*, *Carthami Flos*, *Strychni Semen*, *Eupolyphaga*, *Olibanum*, *Rhizoma Cyperi*, *Rhei Radix et Rhizoma*, *Momordicae Semen*, *Realgar*, *Acori Tatarinowii Rhizoma*, *Phellodendri Cortex*, *Spina Gleditsiae*, *Matrii Sulfas Exsiccatus*, *Indigo Naturalis*	Promoting flow of qi and blood, soften hard masses for eliminating abdominal mass, heat-clearing, and detoxication, removing dampness, and destroying parasites, indicated to cure psoriasis

**Table 3:** Compounds isolated from *Curcumae Rhizoma* and *Curcumae Radix*.

Number	Compounds	Part of plant	Species	References
*Sesquiterpenes*				
(a) Guaiane type				
**1**	Curcumol	Rhizome	*C*.* wenyujin*	[14]
		Radix^a^	*C*.* kwangsiensis*	[15]
**2**	Curcumenol	Rhizome	*C*. *phaeocaulis*	[16]
		Radix	*C*. *wenyujin*	[17]
**3**	Isocurcumenol	Rhizome	*C*. *kwangsiensis*	[14, 15]
		Rhizome	*C*. *phaeocaulis*	[16]
		Radix	*C*. *wenyujin*	[18]
**4**	4-Epicurcumenol	Radix	*C*. *wenyujin*	[18]
**5**	Procurcumenol	Rhizome	*C*. *wenyujin*	[4]
		Radix	*C*. *wenyujin*	[17]
**6**	Isozedoarondiol	Rhizome	*C*. *wenyujin*	[19]
		Radix^a^	*C*. *kwangsiensis*	[15]
**7**	Aerugidiol	Rhizome	*C*. *wenyujin*	[4]
		Rhizome	*C*. *kwangsiensis*	[20]
**8**	Alismoxide	Rhizome	*C*. *wenyujin*	[4]
		Radix^a^	*C*. *kwangsiensis*	[15]
		Radix	*C*. *wenyujin*	[17, 18]
**9**	Guaidiol	Rhizome	*C*. *kwangsiensis*	[21]
**10**	Zedoalactone A	Rhizome	*C*. *wenyujin*	[22]
**11**	Zedoalactone B	Rhizome	*C*. *wenyujin*	[19, 20]
**12**	Zedoalactone C	Rhizome	*C*. *wenyujin*	[22]
		Radix^a^	*C*. *kwangsiensis*	[15]
**13**	Curcumafuranol	Rhizome	*C*. *wenyujin*	[23]
		Rhizome	*C*. *kwangsiensis*	[18, 22]
**14**	Gweicurculactone	Rhizome	*C*. *wenyujin*	[24]
		Radix	*C*. *wenyujin*	[16, 25]
**15**	Zedoalactone D	Rhizome	*C*. *wenyujin*	[26]
		Rhizome	*C*. *kwangsiensis*	[27]
**16**	Zedoalactone E	Rhizome	*C*. *wenyujin*	[26]
		Rhizome	*C*. *kwangsiensis*	[27]
**17**	Zedoalactone F	Rhizome	*C*. *wenyujin*	[26]
**18**	Zedoalactone H	Rhizome	*C*. *wenyujin*	[26]
**19**	(1S,4S,5S,10R)-Zedoarondiol	Rhizome	*C*. *wenyujin*	[19, 26]
**20**	(4S)-4-Hydroxy-gweicurculactone	Rhizome	*C*. *wenyujin*	[28]
**21**	Zedoalactone G	Rhizome	*C*. *wenyujin*	[28]
**22**	(1R,4R,5S,10S)-Zedoalactone B	Rhizome	*C*.* wenyujin*	[28]
**23**	(+)-Zedoalactone A	Rhizome	*C*. *wenyujin*	[28]
**24**	Aeruginolactone	Rhizome	*C*. *wenyujin*	[19]
**25**	1*β*,4*β*,10*β*-Trihydroxy-5*β*H-guai-7(11),8-dien-12,8-olide	Rhizome	*C. wenyujin*	[19]
**26**	4*α*,10*α*-Dihydroxy-1,5,8*β*H-guai-7(11)-en-12,8-olide	Rhizome	*C. wenyujin*	[19]
**27**	4*β*-Hydroxy-5*β*H-guai-1(10),7(11),8-trien-12,8-olide	Rhizome	*C. wenyujin*	[19]
**28**	Isocurcumenol	Rhizome	*C. wenyujin*	[29, 30]
**29**	1*α*,8*α*-Epidioxy-4*α*-hydroxy-5*α*H-guai-7(11),9-dien-12,8-olide	Rhizome	*C. wenyujin*	[4]
**30**	8,9-Seco-4*β*-hydroxy-1*α*,5*β*H-7(11)-guaen-8,10-olide	Rhizome	*C. wenyujin*	[4]
**31**	8*α*-Hydroxy-1*α*,4*β*,7*β*H-guai-10(15)-en-5*β*,8*β*-endoxide	Rhizome	*C. wenyujin*	[4]
**32**	7*β*,8*α*-Dihydroxy-1*α*,4*α*H-guai-10(15)-en-5*β*,8*β*-endoxide	Rhizome	*C. wenyujin*	[4]
**33**	Gweicurculactone	Rhizome	*C. kwangsiensis*	[24]
**34**	9-*Oxo*-neoprocurcumenol	Rhizome	*C. kwangsiensis*	[29, 30]
**35**	Linderazulene	Rhizome	*C. kwangsiensis*	[18, 31]
**36**	Neozedoarondiol	Rhizome	*C. kwangsiensis*	[27]
**37**	Wenyujinin A	Rhizome	*C. wenyujin*	[32]
**38**	Wenyujinin B	Rhizome	*C. wenyujin*	[32]
**39**	Wenyujinin F	Rhizome	*C. wenyujin*	[32]
**40**	Wenyujinin G	Rhizome	*C. wenyujin*	[32]
**41**	Wenyujinin H	Rhizome	*C. wenyujin*	[32]
**42**	Wenyujinin I	Rhizome	*C. wenyujin*	[32]
**43**	Phaeocaulisins A	Rhizome	*C. phaeocaulis*	[33]
**44**	Phaeocaulisins B	Rhizome	*C. phaeocaulis*	[33]
**45**	Phaeocaulisins C	Rhizome	*C. phaeocaulis*	[33]
**46**	Phaeocaulisins D	Rhizome	*C. phaeocaulis*	[33]
**47**	Phaeocaulisins E	Rhizome	*C. phaeocaulis*	[33]
**48**	Phaeocaulisins F	Rhizome	*C. phaeocaulis*	[33]
**49**	Phaeocaulisins G	Rhizome	*C. phaeocaulis*	[33]
**50**	Phaeocaulisins H	Rhizome	*C. phaeocaulis*	[33]
**51**	Phaeocaulisins I	Rhizome	*C. phaeocaulis*	[33]
**52**	Phaeocaulisins J	Rhizome	*C. phaeocaulis*	[33]
**53**	Gweicucrulacotne	Rhizome	*C. kwangsiensis*	[24]
(b) Germacrane type				
**54**	Curdione	Rhizome	*C. wenyujin*	[4]
		Rhizome	*C. kwangsiensis*	[34, 35]
**55**	Neocurdione	Rhizome	*C. wenyujin*	[36]
**56**	(1R,10R)-Epoxy-(−)-1,10-dihydrocurdione	Rhizome	*C. wenyujin*	[14]
		Rhizome	*C. kwangsiensis*	[14]
**57**	Germacrone	Rhizome	*C. kwangsiensis*	[37]
**58**	Furanogermacrene	Rhizome	*C. wenyujin*	[38]
**59**	(4S,5S)-(+)-Germacrone-4,5-epoxide	Rhizome	*C. wenyujin*	[39]
		Rhizome	*C. kwangsiensis*	[14]
**60**	(1S,10S),(4S,5S)-Germacrone-1(10),4(5)-diepoxide	Rhizome	*C. wenyujin*	[39, 40]
**61**	Wenjine	Rhizome	*C. wenyujin*	[39, 40]
		Rhizome	*C. kwangsiensis*	[37]
**62**	Furanodienone	Rhizome	*C. kwangsiensis*	[37]
**63**	Curdionolide A	Rhizome	*C. wenyujin*	[19]
**64**	Curdionolide B	Rhizome	*C. wenyujin*	[19]
**65**	Curdionolide C	Rhizome	*C. wenyujin*	[19]
**66**	6R-Dehydroxylsipanolinolide	Rhizome	*C. wenyujin*	[26]
**67**	Aeruginolactone	Rhizome	*C. wenyujin*	[41]
**68**	(1E,4Z)-8-Hydroxy-6-oxogermacra-1(10),4,7(11)-trieno-12,8-lactone.	Rhizome	*C. wenyujin*	[41]
**69**	(+)-(4S,5S)-Germacrone-4,5-epoxide	Rhizome	*C. wenyujin*	[42]
**70**	(+)-(1S,4S,5S,10S)-Germacrone-1(10)-4-diepoxide	Rhizome	*C. wenyujin*	[42]
**71**	(1E,4E,8R)-8-Hydroxygermacra-1(10),4,7(11)-trieno-12,8-lactone	Rhizome	*C. wenyujin*	[42]
**72**	(1E,4Z)-8-Hydroxy-6-oxogermacra-1(10),4,7(11)-trieno-12,8-lactone	Rhizome	*C. wenyujin*	[42]
**73**	3-Isopropyl-6,10-dimethyl-cyclodec-6-ene-1,4-dione	Rhizome	*C. wenyujin*	[29, 30]
**74**	3-Isopropylidene-6,10-dimethyl-11-oxa-bicyclo-(8.1.0)-undec-6-en-4-one	Rhizome	*C. wenyujin*	[29, 30]
**75**	Epoxide-4-isopropylidene-1,7-dimethyl-11-oxabicyclo-(8.1.0)-undec-6-en-3-one	Rhizome	*C. wenyujin*	[29, 30]
**76**	3-Isopropyl-6,10-dimethyl-cyclodec-6-ene-1,4-dione	Rhizome	*C. wenyujin*	[29, 30]
**77**	Germacrone-1(10),4,7(11)-triepoxide	Rhizome	*C. wenyujin*	[39]
**78**	Curcumalactone	Rhizome	*C. wenyujin*	[14]
**79**	Wenyujinin J	Rhizome	*C. wenyujin*	[32]
**80**	Wenyujinin K	Rhizome	*C. wenyujin*	[32]
**81**	Zederone	Rhizome	*C. kwangsiensis*	[37]
**82**	Isocurcumenol	Rhizome	*C. phaeocaulis*	[16]
**83**	(1S,10S),(4S,5S)-Germacrone-1(10),4-diepoxide	Rhizome	*C. kwangsiensis*	[40]
**84**	Germacrone-l,10-epoxide	Rhizome	*C. kwangsiensis*	[43]
(c) Bisaborane type				
**85**	*αγ*-Turmerone	Radix	*C. wenyujin*	[18]
**86**	Bisacurone	Radix^b^	*C. longa*	[44]
**87**	Curlone	Rhizome	*C. kwangsiensis*	[45]
**88**	Bisacurone A	Rhizome	*C. kwangsiensis*	[46]
		Radix^b^	*C. longa*	[44]
**89**	Bisacurone B	Radix^b^	*C. longa*	[44]
**90**	Bisacurone C	Radix^b^	*C. longa*	[44]
**91**	(6S)-2-Methyl-6-(4-hydroxyphenyl-3-methyl)-2-hepten-4-one	Radix	*C. longa*	[47]
**92**	(6S)-2-Methyl-6-(4-hydroxyphenyl)-2-hepten-4-one	Radix	*C. longa*	[47]
**93**	(6S)-2-Methyl-6-(4-formylphenyl)-2-hepten-4-one	Radix	*C. longa*	[47]
**94**	5-Hydroxyl-ar-turmerone	Radix	*C. longa*	[47]
**95**	Turmeronol B	Radix	*C. longa*	[47]
**96**	Bisabolone	Radix	*C. longa*	[47]
**97**	Bisabolone-4-one	Radix	*C. longa*	[47]
**98**	Turmeronol A	Radix	*C. longa*	[41, 44]
**99**	Turmerone	Radix	*C. wenyujin*	[18]
(d) Eudesmane type				
**100**	Curcolonol	Rhizome	*C. wenyujin*	[38, 45]
		Rhizome	*C. kwangsiensis*	[48]
**101**	Curcodione	Rhizome	*C. wenyujin*	[26]
**102**	Curcolide	Rhizome	*C. wenyujin*	[4, 23]
**103**	Curcolactone	Rhizome	*C. wenyujin*	[27]
**104**	1*β*,4*α*,8*β*-Trihydroxy-5*α*H,10*β*-eudesm-7(11)-en-8,12-olide	Rhizome	*C. wenyujin*	[49]
**105**	4*α*-Hydroxy-8,12-epoxy-5*β*H,10*β*-eudesma-7,11-dien-1,6-dione	Rhizome	*C. wenyujin*	[49]
**106**	(Z)-1*β*,4*α*-Dihydroxy-5*α*,8*β*(H)-eudesm-7(11)-en-12,8-olide	Rhizome	*C. wenyujin*	[49]
**107**	1*β*,4*α*-Dihydroxy-5*α*,8*β*(H)-eudesm-7(11)-en-12,8-olide	Rhizome	*C. wenyujin*	[49]
**108**	Wenyujinlactone A	Rhizome	*C. wenyujin*	[50]
**109**	Neolitamone A	Rhizome	*C. wenyujin*	[50]
**110**	7-Hydroxy-5(10),6,8-cadinatriene-4-one	Rhizome	*C. wenyujin*	[4]
**111**	Curcolactone	Rhizome	*C. kwangsiensis*	[27]
(e) Elemane type				
**112**	Curzerenone	Radix	*C. wenyujin*	[18]
**113**	Hydroxyisogermafurenolide	Rhizome	*C. wenyujin*	[49]
**114**	5-Isopropenyl-3,6-dimethyl-6-vinyl-5,6,7,7*α*-tetrahydro-4H-benzofuran-2-one	Rhizome	*C. wenyujin*	[29, 30]
		Rhizome	*C. kwangsiensis*	[43]
**115**	7*α*-Hydroxy-5-isopropenyl-3,6-dimethyl-6-vinyl-5,6,7,7*α*-tetrahydro-4H-benzofuran-2-one	Rhizome	*C. wenyujin*	[29, 30]
		Rhizome	*C. kwangsiensis*	[43]
**116**	5-Isopropenyl-3,6-dimethyl-6-vinyl-5,6,7,7*α*-tetrahydro-4H-benzofuran-2-one	Rhizome	*C. wenyujin*	[29, 30]
		Rhizome	*C. kwangsiensis*	[43]
**117**	Curzerene	Rhizome	*C. wenyujin*	[51]
		Radix	*C. wenyujin*	[18]
**118**	*β*-Elemene	Rhizome	*C. kwangsiensis*	[52]
		Radix	*C. wenyujin*	[18]
**119**	*γ*-Elemene	Rhizome	*C. kwangsiensis*	[52]
**120**	*δ*-Elemene	Rhizome	*C. kwangsiensis*	[53]
**121**	Curgerenone		*C. phaeocaulis*	[16]
(f) Spironolactone type				
**122**	Curcumalactone	Rhizome	*C. wenyujin*	[4, 51]
(g) Carabrane type				
**123**	Curcumenone	Rhizome	*C. wenyujin*	[49]
**124**	4S-Dihydrocurcumenone	Rhizome	*C. wenyujin*	[49]
		Rhizome	*C. kwangsiensis*	[45]
**125**	Curcarabranol A	Rhizome	*C. wenyujin*	[49]
		Rhizome	*C. kwangsiensis*	[45]
**126**	Curcarabranol B	Rhizome	*C. wenyujin*	[49]
		Rhizome	*C. kwangsiensis*	[45]
(h) Xanthane type				
**127**	Curcumadionol	Rhizome	*C. wenyujin*	[49]
(i) Other types				
**128**	(6R)-Dehydroxysipanolinolide	Rhizome	*C. wenyujin*	[49]
**129**	Wenyujinin C	Rhizome	*C. wenyujin*	[32]
**130**	Wenyujinin D	Rhizome	*C. wenyujin*	[32]
**131**	Wenyujinin E	Rhizome	*C. wenyujin*	[32]
**132**	Difurocumenone	Rhizome	*C. kwangsiensis*	[54]

*Diarylheptanoids*				
**133**	2,3,5-Trihydroxy-1-(3-methoxy-4-hydroxyphenyl)-7-(3,5-dimethoxy-4-hydroxyphenyl)-heptane	Rhizome	*C. kwangsiensis*	[55]
**134**	2,3,5-Trihydroxy-1-(4-hydroxyphenyl)-7-(3,5-dimethoxy-4-hydroxyphenyl)-heptane	Rhizome	*C. kwangsiensis*	[55]
**135**	(3R′,5S′)-3,5-Dihydroxy-1-(4-hydroxy-3-methoxyphenyl)-7-(3,4-dihydroxyphenyl)-heptane	Rhizome	*C. kwangsiensis*	[55]
**136**	rel-(3R,5S)-3,5-Dihydroxy-1-(3,4-dihydroxyphenyl)-7-(4-hydroxyphenyl)-heptane	Rhizome	*C. kwangsiensis*	[55]
**137**	rel-(3R,5S)-3,5-Dihydroxy-1-(4-hydroxy-3-methoxyphenyl)-7-(4-hydroxyphenyl)-heptane	Rhizome	*C. kwangsiensis*	[55]
**138**	rel-(3R,5S)-3,5-Dihydroxy-1-(3-methoxy-4,5-dihydroxyphenyl)-7-(4-hydroxyphenyl)-heptane	Rhizome	*C. kwangsiensis*	[55]
**139**	(5S)-5-Hydroxy-1-(4-hydroxyphenyl)-7-phenyl-3-heptanone	Rhizome	*C. kwangsiensis*	[55]
**140**	(5S)-5-Hydroxy-1-(4-hydroxyphenyl)-7-(3,4-dihydroxyphenyl)-3-heptanone	Rhizome	*C. kwangsiensis*	[55]
**141**	(5S)-5-Hydroxy-1-(4-methoxy)-7-(3,4-dimethoxy)-4-heptanone	Rhizome	*C. kwangsiensis*	[55]
**142**	(5S)-5-Hydroxy-1-(4-hydroxy-3-methoxyphenyl)-7-(4-hydroxyphenyl)-3-heptanone	Rhizome	*C. kwangsiensis*	[55]
**143**	(3R,5R)-3-Acetoxy-5-hydroxy-1,7-bis(4-hydroxyphenyl)-heptane	Rhizome	*C. kwangsiensis*	[55]
**144**	(3R,5R)-3,5-Dihydroxy-1,7-bis(5-hydroxyphenyl)-heptane	Rhizome	*C. kwangsiensis*	[55]
**145**	(3R,5R)-3-Acetoxy-5-hydroxy-1,7-bis(3,4-dihydroxyphenyl)-heptane	Rhizome	*C. kwangsiensis*	[55]
**146**	(3R,5R)-3,5-Dihydroxy-1,7-bis(3,4-dihydroxyphenyl)-heptane	Rhizome	*C. kwangsiensis*	[55]
**147**	(3R,5R)-3,5-Diacetoxy-1-(3,4-dihydroxyphenyl)-7-(4-hydroxyphenyl)-heptane	Rhizome	*C. kwangsiensis*	[55]
**148**	(3R,5R)-3,5-Dihydroxy-1-(3,4-dihydroxyphenyl)-7-(4-hydroxyphenyl)-heptane	Rhizome	*C. kwangsiensis*	[55]
**149**	(3S,5S)-3-Acetoxy-5-hydroxy-1-(3,4-dihydroxyphenyl)-7-(4-hydroxyphenyl)-heptane	Rhizome	*C. kwangsiensis*	[55]
**150**	(3S,5S)-3,5-Dihydroxy-1-(3,4-dihydroxyphenyl)-7-(4-hydroxyphenyl)-heptane	Rhizome	*C. kwangsiensis*	[55]
**151**	(3R,5R)-3-Acetoxy-5-hydroxy-1-(3,4-dihydroxyphenyl)-7-(4-hydroxyphenyl)-heptane	Rhizome	*C. kwangsiensis*	[55]
**152**	(3R,5R)-3,5-Dihydroxy-1-(4-hydroxy-3-methoxyphenyl)-7-(3,4-dihydroxyphenyl)-heptane	Rhizome	*C. kwangsiensis*	[55]
**153**	(3R,5R)-3,5-Diacetoxy-1,7-bis(3,4-dihydroxyphenyl)-heptane	Rhizome	*C. kwangsiensis*	[55]
**154**	(3R,5S)-3,5-Dihydroxy-1,7-bis(4-hydroxyphenyl)-heptane	Rhizome	*C. kwangsiensis*	[55]
**155**	(5S)-5-Hydroxy-1,7-bis(4-hydroxyphenyl)-heptan-3-one	Rhizome	*C. kwangsiensis*	[55]
**156**	(5S)-5-Hydroxy-1-(4-hydroxy-3-methoxy-phenyl)-7-(3,4-dihydroxyphenyl)-heptan-3-one	Rhizome	*C. kwangsiensis*	[55]
**157**	(3S)-1,7-Bis(4-hydroxyphenyl)-(6E)-6-hepten-3-ol	Rhizome	*C. kwangsiensis*	[56]
**158**	(3R)-1,7-Bis(4-hydroxyphenyl)-(6E)-6-hepten-3-ol	Rhizome	*C. kwangsiensis*	[56]
**159**	(3S)-1,7-Bis(4-methoxyphenyl)-(6E)-6-hepten-3-ol	Rhizome	*C. kwangsiensis*	[56]
**160**	(3R)-1,7-Bis(4-methoxyphenyl)-(6E)-6-hepten-3-ol	Rhizome	*C. kwangsiensis*	[56]
**161**	(3S)-1-(3,4-Dihydroxyphenyl)-7-(4-hydroxyphenyl)-(6E)-6-hepten-3-ol	Rhizome	*C. kwangsiensis*	[56]
**162**	(3R)-1-(3,4-Dihydroxyphenyl)-7-(4-hydroxyphenyl)-(6E)-6-hepten-3-ol	Rhizome	*C. kwangsiensis*	[56]
**163**	(3S)-1-(3,4-Dimethoxyphenyl)-7-(4-methoxyphenyl)-(6E)-6-hepten-3-ol	Rhizome	*C. kwangsiensis*	[56]
**164**	(3S)-1-(3,4-Dimethoxyphenyl)-7-(4-methoxyphenyl)-(6E)-6-hepten-3-ol	Rhizome	*C. kwangsiensis*	[56]
**165**	(3S)-3-Acetoxy-1-(3,4-dihydroxyphenyl)-7-(4-hydroxyphenyl)-(6E)-6-heptene	Rhizome	*C. kwangsiensis*	[56]
**166**	(3R)-3-Acetoxy-1-(3,4-dihydroxyphenyl)-7-(4-hydroxyphenyl)-(6E)-6-heptene	Rhizome	*C. kwangsiensis*	[56]
**167**	(3S)-3-Acetoxy-1-(3,4-dimethoxyphenyl)-7-(4-methoxyphenyl)-(6E)-6-heptene	Rhizome	*C. kwangsiensis*	[56]
**168**	(3R)-3-Acetoxy-1-(3,4-dimethoxyphenyl)-7-(4-methoxyphenyl)-(6E)-6-heptene	Rhizome	*C. kwangsiensis*	[56]
**169**	(3S)-1-(3,4-Dihydroxyphenyl)-7-(4-hydroxyphenyl)-heptan-3-ol	Rhizome	*C. kwangsiensis*	[56]
**170**	(3R)-1-(3,4-Dihydroxyphenyl)-7-(4-hydroxyphenyl)-heptan-3-ol	Rhizome	*C. kwangsiensis*	[56]
**171**	(3S)-1-(3,4-Dimethoxyphenyl)-7-(4-methoxyphenyl)-heptan-3-ol	Rhizome	*C. kwangsiensis*	[56]
**172**	(3R)-1-(3,4-Dimethoxyphenyl)-7-(4-methoxyphenyl)-heptan-3-ol	Rhizome	*C. kwangsiensis*	[56]
**173**	(3S)-3-Acetoxy-1-(3,4-dihydroxyphenyl)-7-(4-hydroxy-phenyl)-heptanes	Rhizome	*C. kwangsiensis*	[56]
**174**	(3R)-3-Acetoxy-1-(3,4-dihydroxyphenyl)-7-(4-hydroxy-phenyl)-heptanes	Rhizome	*C. kwangsiensis*	[56]
**175**	(3S)-1-(3,4-Dihydroxyphenyl)-7-phenyl-(6E)-6-hepten-3-ol	Rhizome	*C. kwangsiensis*	[56]
**176**	(3R)-1-(3,4-Dihydroxyphenyl)-7-phenyl-(6E)-6-hepten-3-ol	Rhizome	*C. kwangsiensis*	[56]
**177**	(E)-1,7-Bis(4-hydroxyphenyl)-6-hepten-3-one	Rhizome	*C. kwangsiensis*	[56]
**178**	(E)-1-Bis(4-hydroxyphenyl)-7-phenyl-(6E)-6-hepten-3-one	Rhizome	*C. kwangsiensis*	[56]
**179**	(E)-1,7-Bis(4-hydroxyphenyl)-3-one	Rhizome	*C. kwangsiensis*	[56]
**180**	(E)-1-(4-Hydroxy-3-methoxyphenyl)-7-(4-hydroxyphenyl)-3-one	Rhizome	*C. kwangsiensis*	[56]
**181**	(E)-1,7-Bis(4-hydroxyphenyl)-4,6-heptadiene-3-one	Rhizome	*C. kwangsiensis*	[56]
**182**	(E)-1-(4-Hydroxy-3-methoxyphenyl)-7-(4-hydroxyphenyl)-(4E)-4-hepten-3-one	Rhizome	*C. kwangsiensis*	[56]
**183**	1,5-Epoxy-3-carbonyl-1,7-bis(4-hydroxyphenyl)-4,6-heptadiene	Radix^b^	*C*. *longa*	[57]
**184**	Dihydrocurcumin	Radix^b^	*C*. *longa*	[17]
**185**	Curcumin	Radix^b^	*C*. *longa*	[17]
**186**	Demethoxycurcumin	Radix^b^	*C*. *longa*	[17]
**187**	Bisdemethoxycurcumin	Radix^b^	*C*. *longa*	[17]
**188**	1,7-Bis(4-hydroxyphenyl)-1,4,6-heptatrien-3-one	Radix^b^	*C*. *longa*	[17]
**189**	1,5-Bis(4-hydroxyphenyl)-penta-(1E,4E)-1,4-dien-3-one	Radix^b^	*C*. *longa*	[17]
**190**	1,5-Bis(4-hydroxy-3-methoxyphenyl)-penta-(1E,4E)-1,4-dien-3-one	Radix^b^	*C*. *longa*	[57]

*Other types*				
**191**	Dehydro-1,8-cineole	Rhizome	*C. wenyujin*	[29, 30]
**192**	*p*-Menth-2-ene-1,8-diol	Rhizome	*C. wenyujin*	[29, 30]
**193**	Wenyujinin L	Rhizome	*C. wenyujin*	[32]
**194**	Curcumrinol A	Radix^b^	*C. wenyujin*	[58]
**195**	Curcumrinol B	Radix^b^	*C. wenyujin*	[58]
**196**	Curcumrinol C	Radix^b^	*C. wenyujin*	[58]
**197**	Curcumrinol D	Radix	*C. wenyujin*	[59]
**198**	Curcumrinol E	Radix	*C. wenyujin*	[59]
**199**	Curcumrinol F	Radix	*C. wenyujin*	[16, 56]
**200**	Curcumrinol G	Radix	*C. wenyujin*	[16, 56]
**201**	2-(2′-Methyl-1′-propenyl)-4,6-dimethyl-7-hydroxyquinoline	Radix^b^	*C. longa*	[44]
**202**	Curcumrinol I	Radix	*C. wenyujin*	[18]
**203**	Aurantiamide	Radix	*C. wenyujin*	[60]
**204**	Crotepoxide	Radix^b^	*C. wenyujin*	[17]
**205**	Wenyujinoside	Radix	*C. wenyujin*	[60]
**206**	*β*-Sitosterol	Radix	*C. kwangsiensis*	[61]

^a^Original paper expressed for root.

^b^Original paper expressed for root tuber.
